# RSV is the main cause of severe respiratory infections in infants and young children in Germany - data from the prospective, multicenter PAPI study 2021–2023

**DOI:** 10.1007/s15010-025-02484-1

**Published:** 2025-02-19

**Authors:** Martin Wetzke, Matthias Lange, Cordula Koerner-Rettberg, Alexander Kiefer, Michael Kabesch, Sven Armbrust, Kerim Abdelkhalek, Christiane Lex, Markus Hufnagel, Sebastian Bode, Michael Dördelmann, Michael Lorenz, Stefan Arens, Markus Panning, Holger Köster, Rolf Kramer, Mathieu Bangert, Frank Eberhardt, Grit Barten-Neiner, Christine Happle

**Affiliations:** 1 Pediatric Pneumology, Allergology, Neonatology of Hannover Medical School and German Center for Lung Research, Biomedical Research in End-stage and Obstructive Lung Disease Hannover (BREATH), Hannover, Germany; 2 University Clinic for Pediatrics and Adolescent Medicine (Elisabeth Children’s Hospital), Oldenburg Clinic, Oldenburg, Germany; 3 Children’s Clinic at Marien-Hospital Wesel, Wesel, Germany; 4 Children’s University Clinic of Eastern Bavaria (KUNO), St. Hedwig Clinic of the Brothers of Mercy, University of Regensburg, Regensburg, Germany; 5 Pediatric Clinic, Dietrich-Bonhoeffer-Klinikum, Neubrandenburg, Germany; 6 Klinikum Region Hannover, Children’s Hospital Neustadt am Rübenberge, Neustadt am Rübenberge, Germany; 7 Pediatric Pneumology, University Medicine Göttingen, Göttingen, Germany; 8 Section of Pediatric Infectiology and Rheumatology, Center for Pediatric and Adolescent Medicine, Medical Faculty, University Medical Center, University of Freiburg, Freiburg, Germany; 9 Pediatric Pneumology, University Hospital Ulm, Ulm, Germany; 10 Diako Children’s Hospital Flensburg, Flensburg, Germany; 11 Pediatric Pneumology, University Hospital Jena, Jena, Germany; 12 Pediatric Hospital auf der Bult, Hanover, Germany; 13 Institute of Virology, Faculty of Medicine, University of Freiburg, Freiburg, Germany; 14 Sanofi-Pasteur, Frankfurt am Main, Germany; 15 Capnetz Stiftung, Hannover, Germany

**Keywords:** RSV, Disease burden, Morbidity, Hospitalization, Seasonality

## Abstract

**Background:**

Respiratory syncytial virus (RSV) is one of the main causes of morbidity in infants and young children worldwide. Current data on RSV-associated disease burden in Germany before the introduction of new immunization strategies is lacking.

**Methods:**

The PAPI study is a multicenter, prospective surveillance study of lower respiratory tract infections (LRTI) in children aged ≤ 24 months in Germany.

**Results:**

Data from 1607 children with LRTI hospitalized in twelve German hospitals between September 2021 and May 2023 were analyzed. Among these children, RSV was the most frequently detected pathogen (57.1%), followed by rhino/entero-, metapneumo- and parainfluenza virus. Children with RSV were significantly younger than those with LRTI of other causes (mean of 5.6 ± SD 6.1 vs. mean of 10.1 ± SD 7.3 months, *p* < 0.001) and more frequently affected in their first six months of life. RSV positive children were significantly more likely to develop hypoxemia (61.9% vs. 44.3%, *p* < 0.001) and need for intravenous or enteral fluid supplementation (48.1% vs. 43.1%, *p* = 0.009; 13.2% vs. 5.9%, *p* < 0.001) than those without RSV.

**Conclusion:**

RSV is the dominant pathogen for LRTI-associated hospitalizations in children ≤ 24 months in Germany and associated with a particularly high need for treatment. The ongoing implemented use of RSV immunization according to current recommendations could lead to significant reduction in early childhood morbidity in Germany.

**Supplementary Information:**

The online version contains supplementary material available at 10.1007/s15010-025-02484-1.

## Introduction

Lower respiratory tract infections (LRTIs) are one of the main causes of early childhood morbidity and mortality worldwide [1], with respiratory syncytial virus (RSV) being the most commonly detected pathogen in infants and young children [2, 3]. It is estimated that globally approximately 2% of deaths up to the age of 5 years and around 3.5% of deaths of infants aged 28 days to six months are due to RSV [4]. While the risk of severe RSV disease is particularly high in children with pre-existing conditions such as prematurity or heart defects, the main burden of RSV occurs in children without classic risk factors [5–7]. Although the rate of RSV-associated deaths is low in economically developed countries, the virus also there poses major challenges to healthcare systems, due to the high numbers of affected patients with enormous cumulative morbidity and seasonal treatment costs [4, 8–10]. RSV shows a seasonal occurrence in the Northern hemisphere, typically from December to March [11]. Cases are recorded in the established surveillance systems for respiratory tract infections of the and German federal government agency for disease control and prevention Robert Koch Institute (RKI), but these surveillance programs focus on patients in primary care and do not collect clinical data, so that a clear estimation of RSV-associated morbidity is not possible [12].

There is no active vaccination of infants against RSV. Even though new publications report on the efficacy of the antiviral drug ziresovir in severe RSV bronchiolitis in infants [13, 14] there is as yet no effective use of antiviral or other causal therapeutic agents outside of studies. Novel prevention concepts are based on active vaccination of pregnant women in the third trimester [15] or the application of long-acting monoclonal RSV antibodies (mAb) to newborns and infants before their first RSV season [16]. Since June 2024, the Standing Committee on Vaccination at the Robert Koch Institute (STIKO) has recommended the use of the mAb nirsevimab for primary prevention of RSV disease in all infants in the first year of life, regardless of the presence of a risk constellation [17].

A precise inventory of the RSV-associated disease burden enables the measurement of effects of new prevention programs and adequate adaptation of care structures. The Pediatric Airway Pathogen Incident (PAPI) study was established with the aim of describing the dynamics, frequency and clinical course of viral respiratory tract infections in infants and young children in Germany [18]. In our current analysis, we evaluate the disease burden associated with severe RSV in Germany. We aimed at describing disease severity and treatment demand from children aged ≤ 24 months hospitalized with LRTI and compare morbidity and risk factors in RSV positive versus RSV negative children.

## Methods

### Prospective data collection

PAPI is a multicenter, prospective study analyzing respiratory pathogens and associated disease burden in infants and young children aged ≤ 24 months in Germany. Twelve centers participated in this study (Flensburg Children’s Hospital, Freiburg University Children’s Hospital, Göttingen University Children’s Hospital, Hanover Medical School, Children’s Hospital auf der Bult Hanover, Jena University Children’s Hospital, Neubrandenburg Children’s Hospital, Neustadt am Rübenberge Children’s Hospital, Oldenburg University Children’s Hospital, Ulm University Children’s Hospital, Regensburg University Children’s Hospital, Marien-Hospital Wesel). Inclusion criteria were: age ≤ 24 months, LRTI associated hospitalization with presence of signs and symptoms according to a clear case definition (Suppl. Table 1), written informed consent by caregivers. Exclusion criteria were: hospitalized for more than > 72 h prior to study inclusion, presenting with non-infectious cause for symptoms, prior study inclusion during current season). Recruiting was performed between fall 2021 and spring 2023, from week 39 to week 17. Recruited children received a nasopharyngeal swab for PCR-based diagnosis of the following respiratory viruses (RSV A and B, adenovirus, coronavirus 229E/HKU1/NL63/OC43, SARS-CoV-2, MERS-CoV, bocavirus, metapneumovirus, rhinovirus/enterovirus, influenza A/AH1/H1-2009/AH3/B, parainfluenza 1/2/3/4; GenMark Diagnostics ePlex Respiratory Pathogen Panel 2 (Roche Diagnostics, Rotkreuz, Switzerland). Data were collected pseudonymously in an electronic database.

### Statistical analyses

Analyses were carried out using Microsoft Excel (version 2019), SPSS Statistics (version 29) and GraphPad Prism (version 5). Main variables and information on their scaling and operationalization can be found in the supplement (Suppl. Table 2). Missing values occurred in < 1% for duration of hospitalization, ventilation, and intensive care treatment demand, and < 10% for further variables. Information availability per item is reported in each table. To explore statistical differences in age and sex, we used T or Mann-Whitney-U testing. Subsequently, age and sex adjustment was performed employing binary-logistic for categorical or linear regression for numerical items. The significance level α was set to p 0.05.

### Ethics

The study was approved by the ethics committees of the participating centers (Vote Hannover Medical School, 9442_BO_K_2020 MHH). Legal guardians of each participating child gave their written informed consent.

## Results

**Fig. 1:**
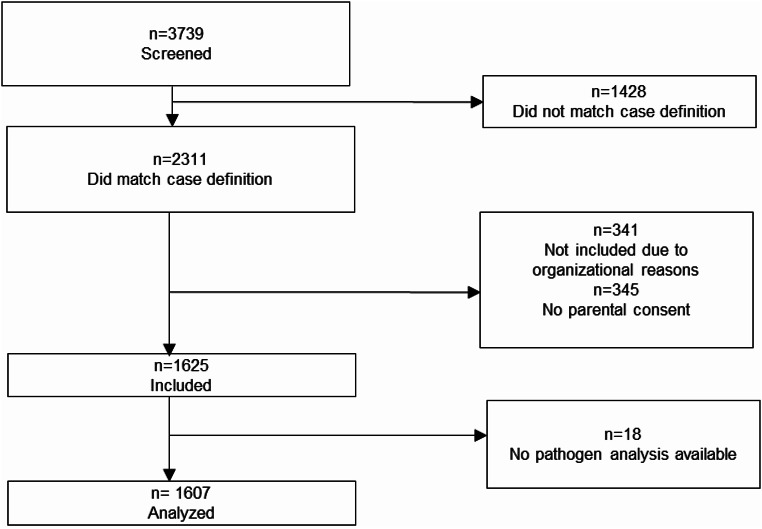
Flowchart with information on screened and included patients

In the two RSV seasons between fall 2021 and spring 2023, a total of 3739 children aged up to 24 months requiring hospitalization were screened for the case definition criteria, and 2311 matched the criteria. Of these, 341 were excluded due to organizational reasons (e.g. study staff was not able to obtain sufficient information or to contact parents), and in 345 cases, no parental consent was granted. A total of 1607 children (536 in season 21/22, 1071 in season 2022/2023) with complete pathogen analysis were included in the analysis (Fig. 1).

**Fig. 2:**
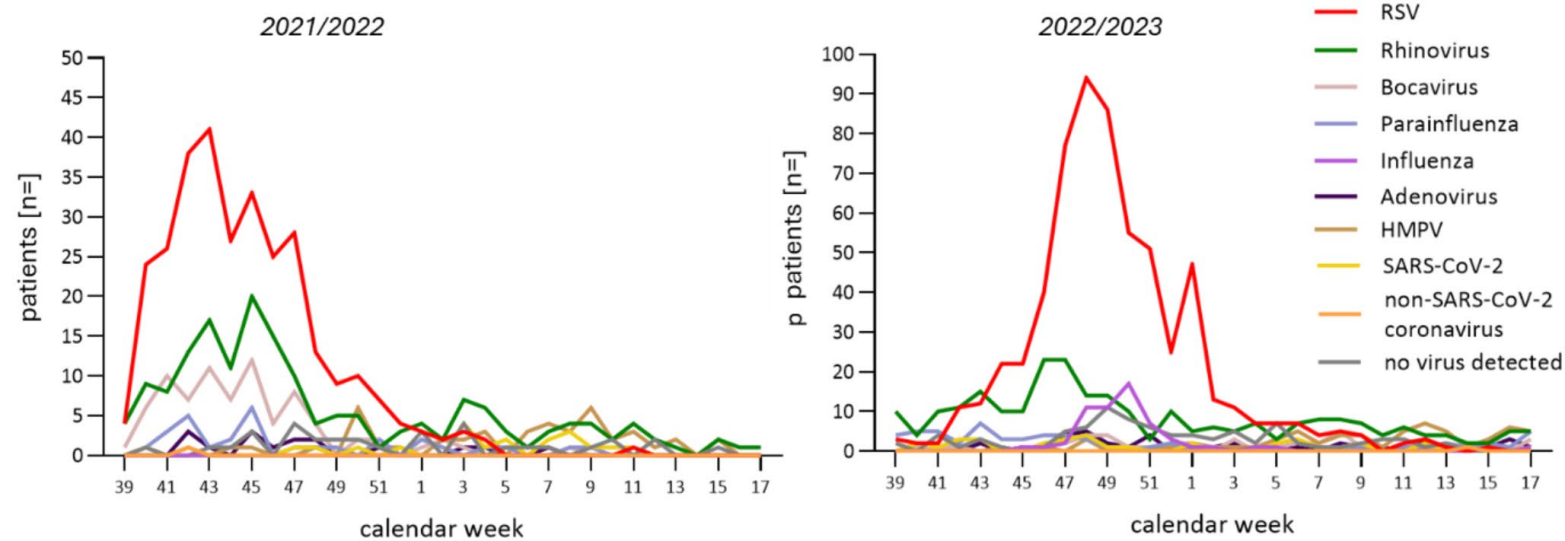
Pathogen distribution by calendar week over the two seasons with RSV as the dominant pathogen in both years

RSV was by far the most frequently detected virus in both seasons and was detected in 918 children (57.1% of the cohort), followed by rhinovirus/ enterovirus (27.8%, Fig. 2, Supp Table 1). 59 children (3,7%) tested influenza positive. In 2021, a clearly premature RSV season (previously typically starting around calendar week 50 [11]) with an activity peak in week 43 occurred, and in the following year a shift of the highest RSV activity towards the end of the year (week 49) could be observed. While RSV-A was the dominant RSV type in the first analysis period (2021/2022: 65.3%, RSV-B: 32.3%; RSV-AB: 2.3%), RSV-B was more common in the second season (2022/2023: 84.6% of all RSV positive cases; RSV-A: 12.8%; RSV-AB: 2.6%; Suppl. Table 3). Children with RSV related LRTI significantly more often displayed coinfections with other viruses than children without RSV (detection of more than one virus type in 28.6% of RSV positive children as compared to 21.4% of RSV negative patients, *p* < 0.001).

**Table 1:** Risk factors and palivizumab prophylaxis in RSV positive versus RSV negative children

	Total	RSV positive	RSV negative	RSV positive vs. negative
Prematurity (< 37th week of pregnancy), (%) [n=]	9.9 [159/1607]	8.6 [79/918]	11.6 [80/689]	*OR 0.8 (95%CI 0.5–1.1* *p* = 0.1
Chronic lung disease of prematurity, (%) [n=]	2 [32/1537]	1.4 [13/897]	2.8 [19/672]	*OR 1.2 (95%CI 0.5–2.9)* *p* = 0.7
(other) chronic lung disease, (%) [n=]	2 [32/1537]	1.4 [13/897]	2.8 [19/672]	*OR 0.6 (95%CI 0.4–0.9)* *p* = 0.02
Heart defect, (%) [n=]	6.3 [99/1583]	5.3 [48/898]	7.5 [51/683]	*OR 1.1 (95%CI 0.6–1.9)* *p* = 0.8
Palivizumab, (%) [n=]	2.5 [37/1492]	0.8 [7/860]	4.7 [30/632]	*OR 0.2 (95%CI 0.1–0.4)* *p* < 0.001

Age/sex-adjusted binary logistic regression analysis

**Fig. 3:**
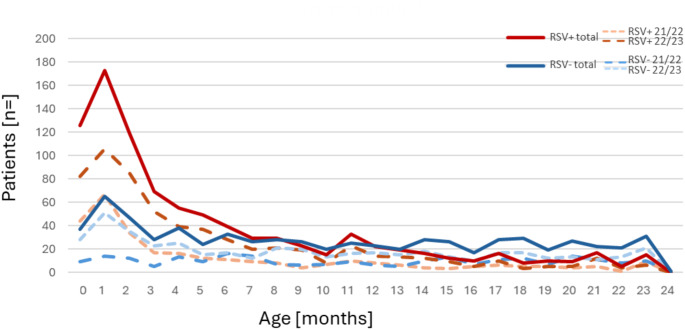
Age distribution among RSV positive and RSV negative patients

The overall mean age within the cohort was 7.5 ± SD 7 months, and patients with RSV associated LRTI were significantly younger than those with respiratory infections due to other pathogens (mean of 5.6 ± SD 6.1 vs. mean of 10.1 ± SD 7.3 months, *p* < 0.001). Accordingly, the proportion of infants recruited within the first six months of life was significantly higher in RSV positive children than in the RSV negative group (Fig. 3 and 68.1% vs. 39.6%; *p* < 0.001). Overall, more boys than girls were included (58.1% male). However, the proportion of boys among RSV positive patients was significantly lower than in the group of patients without RSV detection (RSV positive: 54.3% boys vs. RSV negative 63.1% boys, p0.001). Based on these differences, an adjustment for age and gender was performed for all subsequent analyses.

Upon admission, fever was reported for a significantly smaller proportion of children with than without RSV (59.6% vs. 62.1%, age/sex adjusted *p* = 0.005), and significantly more of RSV positive than RSV negative children presented with rhinitis (83.3% vs. 77.9%, age/sex adjusted *p* = 0.046) and cough (97.7% vs. 94.2%, age/sex adjusted *p* < 0.001). Wheezing was less frequent in RSV positive children than in those without RSV (50.4% vs. 57.5%, adjusted for age and sex *p* = 0.22, unadjusted *p* = 0.005).

Next, we compared the need for treatment in both groups. Children with RSV were hospitalized a median of one day longer than those without (Table 2). This difference was significant before adjustment for age and gender (*p* < 0.001), but no longer significant after this adjustment (Table 2). The cumulative number of hospital treatment days in children with RSV detection during the observation period was 4583 days, whereas children with all other respiratory pathogens together were hospitalized for a total of 3027 days. In the age- and sex-adjusted analysis, children with RSV were significantly more likely to require oxygen supplementation (Table 2). There was also a clear difference between the duration of O_2_ supplementation in children with vs. without RSV detection (in RSV cases a median of 20 h longer, before age/sex adjustment *p* = 0.001), however, after adjustment for age and sex this difference was no longer significant (Table 2).

**Table 2:** Treatment demand in RSV positive versus RSV negative children

	Total	RSV-LRTI	Non-RSV-LRTI	RSV positive vs. negative
Duration of hospital stay, Median days */ IQR;* [n=]	3*/ 4* [1607]	4/ *4* [918]	3/ *3* [689]	*RC -0.53 (95CI -1.1-0.05)* *p* = 0.07
Proportion O_2_ Supplementation (%) [n=]	54.3 872/1605	61.9 [568/918]	44.3 [304/687]	*OR 2.03 (95CI 1.6–2.5)* *p* < 0.001
Duration O_2_ Supplementation, Median days*/ IQR;* [n=]	60/ *74* [555]	68/ *62* [262]	48/ *72* [293]	*RC -6.3 (95CI -16.8-4.2)* *p* = 0.2
Non-invasive ventilation (NIV), (%) [n=]	8.0 [128/1607]	8.5 [78/918]	7.3 [50/689]	*OR 0.8 (95CI 0.6–1.1)* *p* = 0.4
Invasive ventilation (IV), (%) [n=]	1.3 [21/1607]	1.2 [11/918]	1.6 [10/689]	*OR 0.8 (95CI 0.3-2)* *p* = 0.6
Stomach tube, (%) [n=]	10.0 [146/1455]	13.2 [110/830]	5.9 [37/624]	*OR 1.7 (95CI 1.2–2.6)* *p* < 0.001
Intravenous fluid administration, (%) [n=]	46.0 [668/1454]	48.1 [399/830]	43.1 [269/624]	*OR 1.4 (95CI 1.1–1.7)* *p* = 0.009
Administration of antibiotics, (%) [n=]	27.5 [400/1454]	25.7 [213/830]	30 [187/624]	*OR 1.0 (95CI 0.8–1.3)* *p* = 0.9
Administration of inhaled ß mimetics, (%) [n=]	67.1 [976/1454]	61.7 [512/830]	74.4 [464/624]	*OR 0.8 (95CI 0.6–0.9)* *p* = 0.02

Age/sex-adjusted linear regression analysis for durations of hospital stay and oxygen supplementation, binary logistic regression analysis for all other items; RC: regression coefficient for quantitative/numerical items; OR: Odd’s ratio for categorical items)

The fractions of children with non-invasive and invasive ventilation (NIV, IV) did not differ significantly between the two groups, neither before nor after adjustment for age and sex (Table 2, p-values without age/sex adjustment: NIV *p* = 0.4; IV *p* = 0.7). The demand for intensive care treatment occurred more frequently in RSV positive children than in patients without RSV (proportion 11.5% in RSV positive vs. 8% in RSV negative children, without age/sex adjustment *p* = 0.01, not significant after age/sex adjustment *p* = 0.41). The duration of intensive care treatment was approximately the same in both groups and amounted to a median of 5 days in RSV negative and positive children (significance without age/sex adjustment *p* = 0.8, with adjustment *p* = 0.16). No deaths were observed in either group over the entire observation period.

Children with RSV significantly more often required fluid supplementation (intravenous or by gastric tube, Table 2). Antibiotic treatment was provided less frequently to patients with RSV associated LRTI, but this difference was not significant between the two groups (Table 2, p-value without age/sex adjustment 0.75). Inhaled ß-mimetics, however, were administered significantly more often to patients with LRTI due to other pathogens than to those with RSV (Table 2).

With regard to risk factors, the proportion of preterm infants was slightly higher in the group of RSV negative children, but overall not significantly different between the groups (Table 1; significance without age/sex adjustment *p* = 0.051). The rates of children with heart defects and chronic lung disease of prematurity (CLD due to bronchopulmonary dysplasia) were also lower in RSV negative patients, but not significantly different between the two groups (Table 1, significance without age/sex adjustment for heart defects *p* = 0.7; for CLD *p* = 0.8). However, there was a significantly higher proportion of patients with other chronic lung diseases such as airway hyperresponsiveness among RSV negative children (Table 1).

The presence of older siblings in the household was reported more frequently in the group of children with RSV detection than in the RSV negative group (without age/sex adjustment *p* = 0.01, 72.7% vs. 66.3%, after adjustment *p* = 0.5). Significantly lower proportions RSV positive than RSV negative children had attended daycare (15.8% vs. 24.6%, before adjustment for age and sex *p* < 0.001, after adjustment *p* = 0.01), and significantly fewer of them had received RSV prevention with palivizumab (Table 1). Newer RSV prevention drugs or maternal vaccinations were not reported for any of the cases.

## Discussion

The here presented data illustrates that RSV is the main cause of LRTI-associated hospitalizations in the first two years of life in Germany and that affected children display a particularly high degree of morbidity and treatment demand. This finding is in line with numerous studies from other countries that describe RSV as a major cause of severe respiratory infections in infants and young children [4, 5, 9, 19].

After lifting the pandemic-associated non-pharmacological preventive measures, which had led to a virtual absence of the 2020/2021 RSV season [18], our current data show an extra-seasonal occurrence of RSV with clearly increasing case numbers as early as week 39 in summer 2021. In the second year of the here presented analysis, RSV activity started early again, but with a shift towards later of the year when typical pre-pandemic RSV seasons had begun. Similarly to other reports, our data documents the strong impact of the pandemic on non-SARS-CoV-2 pathogens such as RSV [20–22].

The signs and symptoms associated with RSV infections in our cohort (rhinitis, cough, fewer occurrence of fever and wheezing than in RSV negative children) as well as the lower rates of treatment with antibiotic agents or inhaled ß mimetics when compared with LRTI due to other pathogens are in line with previous reports [23, 24]. Our results clearly demonstrate the high morbidity associated with RSV infections in children < 24 months of age. Their therapy demand was significantly higher than that of children with LRTIs due to other pathogens. In an age and sex-adjusted analyses, the rates of need for oxygen supplementation and enteral or intravenous fluid supplementation were significantly higher in the RSV positive subcohort. Also, further disease severity parameters such as duration of hospitalization or the demand for non-invasive ventilation were higher in children with RSV. However, these latter differences were not significant after adjustment for age and sex. Other studies have also shown that RSV is associated with significantly higher morbidity in infants and young children than other respiratory pathogens [25]. In our cumulative analysis, children with RSV required a 1.5 times more inpatient hospital days than children with LRTI due to infection with all other pathogens combined. This strong impact of a singular pathogen explains the seasonal stress on pediatric inpatient admission capacities when RSV season starts, which has been documented across many countries [22, 26, 27].

The high RSV-associated treatment demand is particularly relevant in context of the newly approved preventive measures. Since June 2024, the German Standing Committee for vaccination has recommended the use of nirsevmab in all newborns and infants before their first RSV season to prevent RSV related disease [17]. Our data suggests that effective RSV prevention could lead to fundamentally reduced morbidity and a significant reduction in seasonal hospitalization rates in German children’s hospitals. In Spain, universal availability of nirsevimab for all infants (depending on the region, coverage rates of 79–99% of all infants were reached), was associated with a 70–90% decrease in RSV cases requiring oxygen support [28, 29]. Furthermore, recent real-world studies from the US, Spain, and France demonstrated an effectiveness against RSV-associated hospitalization of 90%, 82%, and 83%, respectively [29–31]. This, together with our data strongly support the notion the German Standing Committee for vaccination’s position for widespread implementation of preventive measures to reduce the burden of RSV disease: since the season 2023/24 universal RSV prophylaxis in infants has been introduced to the national immunization program [17]. Official recommendations for RSV prevention through maternal vaccination during the third trimester may follow [15]. In addition to prevention strategies, our data may also facilitate decision making regarding the application of effective antiviral drugs for the treatment of children with RSV disease, once these become broadly available [13].

As a side note, we found only few cases of influenza infections within our cohort. While maternal influenza vaccination is promoted for pregnant women in Germany [32], annual influenza vaccinations– in contrast to many other countries [33]– are not part of German regular pediatric vaccination programs. As our data show, influenza was not a highly prevalent pathogen leading to LRTI associated hospitalizations in the age group below two years in the analyzed years. However, our study was not designed to evaluate vaccination regimens against influenza, a pathogen associated with severe disease in children [34] and shifted seasonality after the COVID-19 pandemic [35].

Our study has important limitations. Although data collection was performed prospectively, systematically and in multicenter fashion, the hospitals participating represent only a small fraction of German clinics. Due to the design of our study, extra-seasonal RSV circulation in early and mid summer, which was outside the observation period of the study, may have been missed [11]. The fact that around 30% of children meeting this study´s case definition were not included due to organizational reasons and/or lacking parental consent may have introduced a selection bias. Furthermore, our analyses focused on hospitalized children under two years of age. However, RSV is also an important respiratory pathogen for older children, adults and seniors, as well as in ambulatory care settings [36–39]. Future analyses by the PAPI team will focus on the impact of new prevention measures in upcoming seasons and on the ambulatory care sector.

In summary, we here present the first comprehensive, prospectively collected, and current data on the RSV associated disease burden in hospitalized children under two years of age in Germany. Our results show that RSV is the main pathogen in severe respiratory infections in this age group and illustrate the high need for prevention and therapy in RSV infected infants. We hope that our data on helps to implement and monitor prevention and care structures accordingly.

## Electronic supplementary material

Below is the link to the electronic supplementary material.

Supplementary Material 1

## Data Availability

The data on which the study is based may be made available to third parties with the consent of the PAPI Study Board.
