# Supplementary material for: RSV is the main cause of severe respiratory infections in infants and young children in Germany - data from the prospective, multicenter PAPI study 2021–2023
Source: Infection. 2025 Feb 19;53(5):1715–23. doi: 10.1007/s15010-025-02484-1 (PMC12460574; doi:10.1007/s15010-025-02484-1)
Supplement: Supplementary file 1 — Supplementary Material 1 [file 15010_2025_2484_MOESM1_ESM.docx]

**Supplement**

***Supplementary Table 1: Case definition of lower respiratory tract infection (LRTI). Patients with at least one symptom from both groups were included.***

| *Symptom group A* | *Symptom group B* |
| --- | --- |
| *Fever >38 °C*  *Cough*  *Nasal congestion*  *Rhinorrhea/ Coryza*  *Sore throat* | *Wheezing*  *Crackles or Rales*  *Diminished breath sounds*  *Shortness of breath, rapid or shallow breathing*  *Hypoxemia (O_2_ saturation below 92%)* |

***Supplementary Table 2: Information on scaling and operationalization of variables***

|  | ***Operationalization*** | ***Scale level*** |
| --- | --- | --- |
| **Demographic data, age** | Age s | Numerical, months |
| **Demographic data, sex** | Male, female | categorical, months |
| **Disease severity, treatment demand** | Duration of hospital stay | Numerical, days |
| **Disease severity, treatment demand** | Proportion O2 Supplementation | Categorical (Yes/No;%) |
| **Disease severity, treatment demand** | Duration O2 Supplementation | Numerical, days |
| **Disease severity, treatment demand** | Non-invasive ventilation (NIV) | Categorical (Yes/No;%) |
| **Disease severity, treatment demand** | Invasive ventilation (IV) | Categorical (Yes/No;%) |
| **Disease severity, treatment demand** | Administration of stomach tube | Categorical (Yes/No;%) |
| **Risk factors** | Prematurity (<37 weeks of pregnancy) | Categorical (Yes/No;%) |
| **Risk factors** | Chronic lung disease of prematurity | Categorical (Yes/No;%) |
| **Risk factors** | Heart defect | Categorical (Yes/No;%) |
| **Risk factors** | Palivizumab administration | Categorical (Yes/No;%) |
| **Risk factors** | Siblings living in the household | Categorical (Yes/No;%) |
| **Risk factors** | Attending daycare | Categorical (Yes/No;%) |

***Supplementary Table 3: Number of children with specific virus detection***

|  | 2021/2022 (n) |  | 2022/2023 (n) |  | total (n) |
| --- | --- | --- | --- | --- | --- |
| Influenza A virus | 0 |  | 4 |  | 4 |
| Influenza A virus A/H1N1 | 0 |  | 1 |  | 1 |
| Influenza A virus A/H1N1(pdm09) | 0 |  | 0 |  | 0 |
| Influenza A virus A H3N2 | 0 |  | 47 |  | 47 |
| Influenza B virus | 0 |  | 7 |  | 7 |
| Total influenza | 0 |  | 59 |  | 59 |
| Respiratory syncytial virus A | 196 |  | 79 |  | 275 |
| Respiratory syncytial virus B | 97 |  | 523 |  | 620 |
| Respiratory syncytial virus A/B (co-infection) | 7 |  | 16 |  | 23 |
| Total respiratory syncytial virus | **300** |  | **618** |  | **918** |
| Human metapneumovirus A/B | 45 |  | 63 |  | 108 |
| Parainfluenza virus 1 | 0 |  | 18 |  | 18 |
| Parainfluenza virus 2 | 14 |  | 15 |  | 29 |
| Parainfluenza virus 3 | 6 |  | 24 |  | 30 |
| Parainfluenza virus 4 | 11 |  | 4 |  | 15 |
| Total parainfluenza | 30 |  | 60 |  | 90 |
| Human coronavirus HKU1 | 2 |  | 5 |  | 7 |
| Human coronavirus NL63 | 2 |  | 9 |  | 11 |
| Human coronavirus 229E | 3 |  | 2 |  | 5 |
| Human coronavirus OC43 | 15 |  | 31 |  | 46 |
| Total endemic coronaviruses | 22 |  | 46 |  | 68 |
| SARS-CoV-2 | 20 |  | 35 |  | 55 |
| Human rhinovirus/enterovirus | 172 |  | 274 |  | 446 |
| Adenovirus | 16 |  | 42 |  | 58 |
| Bocavirus | 79 |  | 45 |  | 124 |
| No virus detection | 34 |  | 92 |  | 126 |

*Co-infections are listed separately for RSV-AB only, therefore more virus detections than children in the cohort are listed.*
